# Low-Error Soil Moisture Sensor Employing Spatial Frequency Domain Transmissometry

**DOI:** 10.3390/s22228658

**Published:** 2022-11-09

**Authors:** Tadaomi Saito, Takahiro Oishi, Mitsuhiro Inoue, Sachio Iida, Norihito Mihota, Atsushi Yamada, Kohei Shimizu, Satoru Inumochi, Koji Inosako

**Affiliations:** 1 Faculty of Agriculture, Tottori University, 4-101 Koyama-Minami, Tottori 680-8553, Japan; 2 Sony Group Corporation, 1-7-1 Konan Minato-ku, Tokyo 108-0075, Japan; 3 Department of Dryland Science, Graduate School of Sustainability Science, Tottori University, 4-101 Koyama-Minami, Tottori 680-8550, Japan; 4 United Graduate School of Agricultural Sciences, Tottori University, 4-101 Koyama-Minami, Tottori 680-8553, Japan

**Keywords:** dielectric probe, time domain reflectometry (TDR), time domain transmissometry (TDT), frequency domain reflectometry (FDR), saline soil, arid region

## Abstract

A new type of soil moisture sensor using spatial frequency domain transmissometry (SFDT) was evaluated. This sensor transmits and receives ultrawideband (1 to 6 GHz) radio waves between two separated antennas and measures the propagation delay time in the soil related to the dielectric constant. This method is expected to be less affected by air gaps between the probes and the soil, as well as being less affected by soil electrical conductivity (EC), than typical commercial sensors. The relationship between output and volumetric water content (θ), and the effects of air gaps and EC were evaluated through experiments using sand samples and the prototype SFDT sensor. The output of the SFDT sensor increased linearly with θ and was not affected by even a high salt concentration for irrigation water, such that the EC of the pore water was 9.2 dS·m^−1^. The SFDT sensor was almost unaffected by polyethylene tapes wrapped around the sensor to simulate air gaps, whereas a commercially available capacitance sensor significantly underestimated θ. Theoretical models of the SFDT sensor were also developed for the calibration equation and the air gaps. The calculation results agreed well with the experimental results, indicating that analytical approaches are possible for the evaluation of the SFDT sensor.

## 1. Introduction

Soil water content is one of the most important hydrologic variables that affects surface runoff, infiltration, evaporation, and transpiration. Nondestructive monitoring methods of soil water content are desired for environmental evaluation, precision agriculture, and natural resource management. In recent years, dielectric soil moisture sensors or probes have been widely used for nondestructive determination of volumetric soil water content ($\theta :\;m^{3}\;m^{-3}$). Dielectric moisture sensors output electrical signals depending on the apparent dielectric constant (*ε*) of a soil–water–air mixture. The large difference in dielectric constant between that of dry soil (2–5) and that of pure water (about 80) is used to estimate soil water content [1].

Various methods have been proposed for measuring the dielectric constant of a material. The transmission line method has been used to measure *ε* of a material by cutting and inserting the material into the cross-section of a coaxial line or a waveguide, and then calculating the complex permittivity and permeability from the reflection and transmission coefficients [2,3]. In the free-space method, *ε* of a sample fixed between two horn antennas was measured without using a coaxial line or waveguide [4,5]. Other methods for measuring *ε* are the coaxial probe method, resonant cavity method and capacitor method [6]. However, these methods were laboratory measurements with processed samples. Therefore, it is difficult to apply them to in situ soil moisture measurements.

Well-known methods to estimate *ε* in the soil include time domain reflectometry (TDR), time domain transmissometry (TDT), capacitance techniques, and frequency domain reflectometry (FDR). In these methods, ε is estimated through analysis of reflection/transmission of electromagnetic waves along rods or probes. TDR determines *ε* by measuring the delay in time between the incident and reflected electromagnetic pulses, which propagate along a parallel wave guide, in the form of probes or conductors [1]. TDT is similar to TDR, but it measures the transmission, rather than reflection, of electromagnetic pulses along a looped or closed-circuit rod. TDR/TDT sensors are considered a very reliable and accurate method for determining θ. However, their use in agriculture has been limited by their high cost and the requirement for complex wave form analysis to estimate soil moisture [7]. The high cost of TDR sensors has led to the development of alternative lower-cost, lower-frequency (10–150 MHz) capacitance technique and FDR sensors that do not rely on complicated waveform analysis [7]. Such capacitance sensors consist of a pair of electrodes that form a capacitor with the soil as the dielectric. There are several ways to measure the capacitance that is related to *ε*, including the use of capacitor reactance to form a voltage divider, constructing an RC oscillator where the capacitance is calculated from the frequency, or measuring the capacitance through charging the capacitor and discerning the charging time [8]. FDR is similar to the capacitive technique; it uses swept frequency (collecting the data over a wide range of frequencies) and determines *ε* from the frequency variations of electromagnetic pulses [1]. Owing to the lower operating frequencies of capacitance and FDR sensors, these are more prone to error from soil texture [9,10], electrical conductivity (EC) [8,11,12], and temperature [12,13,14] than TDR sensors [15]. FDR sensors also require careful installation to avoid air gaps between the sensor and the soil, because it is vital to have a good contact between the sensor and soil to ensure reliable measurements [16]. In this paper, we focus on the effects of air gaps and EC on the output of dielectric moisture sensors.

Air gaps between the rods and the soil often occur when conventional sensors are inserted into soil, which cause large errors in the measured values. Thus, a soil moisture sensor with a reduced air gap effect is desired for accurate soil moisture measurement. Conventional dielectric sensors measure *ε* of the soil in the vicinity of the rod. Because the dielectric constant of the air gap is 1, the measured results differ significantly from *ε* of the soil. Bore et al. [17] reported that any local non uniformities around the TDR rod have a great effect on the permittivity measurement because most of the energy of the electric field is located in the vicinity of the TDR rod. Another study confirmed that measurement error increases in proportion to the thickness of the air gap [18]. In addition, in the case of a capacitance sensor, an installation method in which the rods and the soil were in close contact was also recommended because of the variation of capacitance caused by air gaps around the rods [19].

Another study investigated a soil moisture sensor with a small influence of EC for the case when fertilizers were applied to crops or in drylands where salt accumulation was likely to occur. It was reported that *ε* in the soil below 1 GHz varied with EC due to Maxwell–Wagner polarization, and this effect could be reduced by measuring *ε* in the microwave band above 1 GHz [20]. However, conventional soil moisture sensors measure *ε* below 1 GHz, such as the TEROS12 capacitance sensor (METER Group, Inc., Pullman, WA, USA) which uses 70 MHz and the CS616 TDR sensor (Campbell Scientific, Inc., Logan, UT, USA) which uses 70 MHz and harmonics thereof [21,22]. Incidentally, if the effect of EC on sensor output can be reduced, the temperature dependence of the sensor output can also be reduced because EC is temperature-dependent [14,23].

In order to meet these requirements, the Sony Group Corporation (Sony) developed a new type of soil moisture sensor that is less susceptible to air gaps and EC than conventional sensors. The sensor is unique in that it consists of a probe with two separated antennas. This sensor transmits and receives ultrawideband (1 to 6 GHz) radio waves between two antennas and measures the propagation delay time in the soil that is related to the dielectric constant. We named this method spatial frequency domain transmissometry (SFDT). There is a frequency domain transmissometry (FDT) sensor using some frequencies with the transmission lines [24]. However, the waves are swept on the transmission lines and affected by air gaps the same as the TDT sensor because most of the energy of the electric field is located in the vicinity of the transmission lines. The SFDT sensor is expected to be less affected by air gaps because the measured *ε* is the volume-weighted average of the dielectric constant of the soil and air gaps between the separated antennas. In addition, the effect of EC is small because the frequency band used is greater than 1 GHz.

The objectives of this study were as follows: (i) introduction of the measurement principle of the SFDT sensor, (ii) moisture calibration through experiments using sand, (iii) evaluation of the effect of EC and air gaps on sensor outputs, and (iv) comparison between theoretical models of the SFDT sensor and the experimental results. For (ii) and (iii), comparisons were also made with a commercially available capacitance sensor (TEROS12).

## 2. Methods

### 2.1. Spatial Frequency Domain Transmissometry

**Figure 1:**
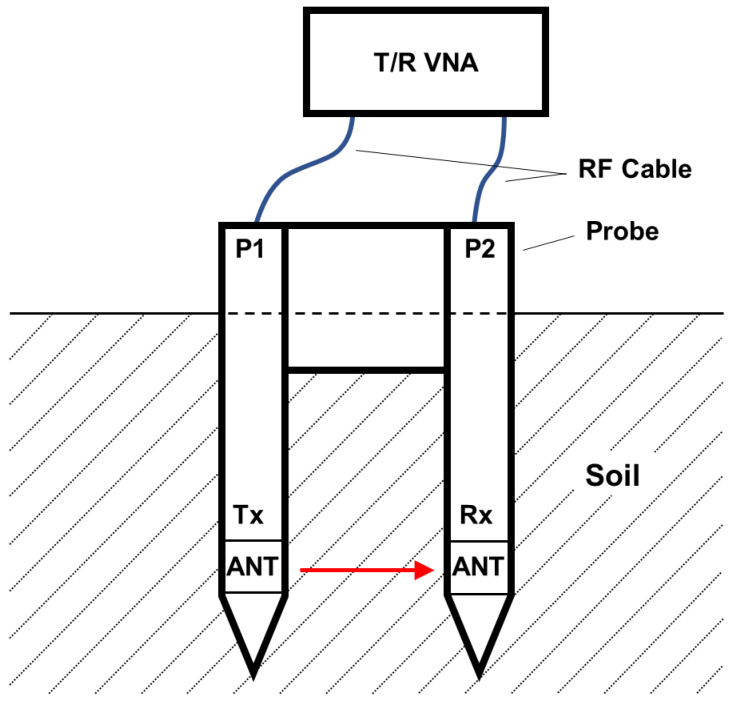
Schematic illustration of the SFDT sensor. Port 1 and Port 2 are expressed as P1 and P2, and the antenna is expressed as ANT.

The SFDT sensor consists of a probe with two antennas, two RF cables, and a transmission/reflection vector network analyzer (T/R VNA) which measures S-parameters ($S_{11}$, $S_{21}$) of 1 to 6 GHz in the same frequency steps at all θ. Figure 1 shows a schematic illustration of the SFDT sensor.

The transmit (Tx) antenna was connected to Port 1 (P1), and the receive (Rx) antenna was connected to Port 2 (P2) on the probe. $S_{11}$ is the reflection coefficient of P1, and $S_{21}$ is the forward transmission coefficient from P1 to P2. To obtain the propagation time between the two antennas, $S_{11}$ and $S_{21}$ were subjected to an inverse fast Fourier transform (IFFT) to calculate $t_{11}$ and $t_{21}$, corresponding to the peak impulse response times of $S_{11}$ and $S_{21}$. $t_{11}$ is the time from P1 to P1 via the Tx ANT, and $t_{21}$ is the time from P1 to P2. The T/R VNA was calibrated on RF connectors, and that position became the calibration plane of P1 and P2. When the path from P1 to the Tx antenna and the path from P2 to the Rx antenna are the same length, the propagation delay time ($t_{pd}$) between the two antennas is expressed as

(1) $$t_{pd}=t_{21}-t_{11}.$$

Aging variation is reduced by Equation (1) because the lengths of the two RF cables expand or shrink by the same amount. The antennas were covered with a plastic housing to stabilize the electric field, since $t_{11}$ should be constant at any θ.

The frequency range of 1 to 6 GHz was chosen considering the propagation characteristics of the soil. If the frequency range was infinite, the desired wave and the wave form obstacles could be completely separated on the time axis. However, above 6 GHz, it is hard to detect the signal due to the loss caused by water, while, below 1 GHz, $t_{pd}$ is affected by Maxwell–Wagner polarization.

If the antennas are in direct contact with the soil, the apparent dielectric constant of the soil ($\varepsilon_{a}$) is defined by

(2) $$\varepsilon_{a}={\left(\frac{ct_{pd}}{d_{0}}\right)}^{2},$$

where $d_{0}$ is the distance between the two antennas, and c is the speed of light. It is also known that $\varepsilon_{a}$ can be expressed as a function of θ. According to Birchak’s empirical model [25], the dielectric constant of a mixture ($\varepsilon_{mix}$) is

(3) $$\varepsilon_{mix}{}^{\alpha }=\sum_{i=1}^{n}{\left(\varepsilon_{i}\right)}^{\alpha }v_{i},$$

where α is an empirical constant, $\varepsilon_{i}$ is the dielectric constant of the i-th material, $v_{i}$ is the volume fraction of the i-th material, and n is the number of phases. Applying the four-phase model to Equation (3), Dobson et al. [26] showed that the apparent dielectric constant of the soil ($\varepsilon_{a}$) is given by

(4) $$\varepsilon_{a}{}^{\alpha }=\left(1-\varphi \right)\varepsilon_{soil}{}^{\alpha }+\left(\theta -\theta_{bw}\right)\varepsilon_{fw}{}^{\alpha }+\theta_{bw}\varepsilon_{bw}{}^{\alpha }+\left(\varphi -\theta \right)\varepsilon_{air}{}^{\alpha },$$

where $\theta_{bw}$ is the volumetric water content of bond water, φ is the porosity of the dry soil, $\varepsilon_{soil}$ is the dielectric constant of the dry soil particle, $\varepsilon_{fw}$ is the dielectric constant of free water, $\varepsilon_{bw}$ is the dielectric constant of bond water, and $\varepsilon_{air}$ is the dielectric constant of air. $t_{pd}$ is expressed from Equations (2) and (4) as

(5) $$t_{pd}=\frac{d_{0}}{c}{\left[\left(1-\varphi \right)\varepsilon_{soil}{}^{\alpha }+\left(\theta -\theta_{bw}\right)\varepsilon_{fw}{}^{\alpha }+\theta_{bw}\varepsilon_{bw}{}^{\alpha }+\left(\varphi -\theta \right)\varepsilon_{air}{}^{\alpha }\right]}^{\frac{1}{2\alpha }}.$$

The calibration equation as a function of θ was prepared by measuring $t_{pd}$ using Equation (5). Because the calibration equation of $\varepsilon_{a}$ and θ had low linearity, the calibration equation of $t_{pd}$ and θ was used in this study. Note that Equation (5) is applicable to the TDR sensor, where $d_{0}$ is twice the length of the TDR rods, and $t_{pd}$ is the round-trip time.

### 2.2. Water Content Calibration Using Sand

The relationship between water content and output values of the SFDT sensor was investigated using sand with different water content. The results were compared with the commercially available capacitance sensor TEROS12.

#### 2.2.1. SFDT Sensor System

**Figure 2:**
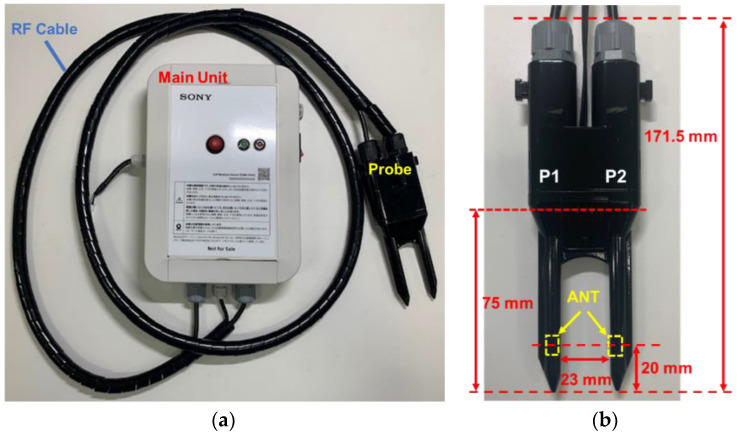
Prototype SFDT sensor: (**a**) SFDT sensor system and (**b**) SFDT sensor probe.

The prototype SFDT sensor is shown in Figure 2.

The prototype consists of the main unit, the probe, and the RF cables that connect the main unit to the probe. The T/R VNA, its signal processing circuit, and the external data communications device were installed in the main unit. S-parameters acquired by the T/R VNA were converted into the propagation delay time by the signal processing circuit in the main unit, and the output data were transmitted to the cloud or a smartphone by the communications device. The raw output data related to the *ε* were propagation delay times in 1 ps steps (θ~0.002 m^3^ m⁻^3^). The measurement time including calculation was approximately 30 s, because a wide frequency range of 1 to 6 GHz was measured and processed. The antennas were located at 20 mm from the tip of the probe, and the distance between the probe rods was 23 mm. The probe was designed to be buried in the soil to a depth of 75 mm from the tip. The prototype was tested to confirm that the electromagnetic field strength at a distance of 3 m from the Tx antenna was less than 35 μV $m^{-1}$ in the air and satisfied the relevant extremely low power radio station regulations of Japan [27].

The SFDT method is similar to the free-space method [4,5]; however, the free-space method is a laboratory measurement with a processed sample and cannot measure in situ soil moisture. The structure around the antenna is also different, since, in the free-space method the wave propagates though the air and the electric field near the antenna is stable, while, in a soil moisture sensor, the waves propagate in the soil. Therefore, the electric field around the antenna needs to be stabilized. Note that the antenna of the SFDT sensor was not simple a horn antenna covered with a plastic housing.

#### 2.2.2. TEROS12 Capacitance Sensor

TEROS12 sensors (METER Group, Inc., Pullman, WA, USA) employ the capacitance method and are widely used low-cost soil moisture sensors. In addition to water content, TEROS12 sensors can measure bulk EC and temperature. The oscillation frequency is 70 MHz. TEROS12 sensors have three rigid steel needles (55 mm in length and 3.2 mm in diameter). The data were collected by connecting to a ZL6 logger (METER Group, Inc., Pullman, WA, USA). Users can choose either θ or raw value as the output. In this study, the raw value was employed as the output value; however, because the original raw value had a large number of digits, the raw value divided by 1000 (R_out_) was used as the output from the TEROS12 sensor.

#### 2.2.3. Calibration Experiments Using Distilled Water

**Figure 3:**
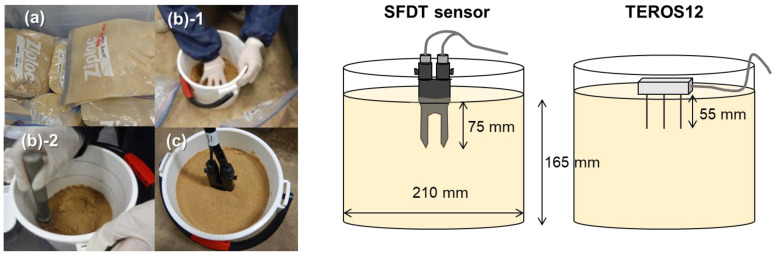
Pictures and schematic diagrams of the experiments: (**a**) moisture-adjusted samples in the plastic bags; (**b**) 1,2 uniform filling of the samples; (**c**) sensor insertion.

Calibration experiments were conducted in a laboratory at a constant room temperature (20 °C). Pictures and schematic diagrams of the experiments are shown in Figure 3. Toyoura sand (Toyoura Keiseki Kogyo Co., Ltd., Yamaguchi, Japan), which comprises 100% sand, was used in the calibration experiments. Sand samples were prepared by adjusting the moisture content in 10 steps from air-dried to saturated using distilled water. The moisture content of each sand sample was adjusted by thoroughly mixing the sand and water in plastic bags. The moisture-controlled sand samples were filled as uniformly as possible (average dry bulk density: 1.40 Mg·m^−3^) into containers (sample height: 165 mm, diameter: 210 mm) whose volumes were sufficiently larger than the sensing volumes of the sensors. The SFDT sensor and TEROS12 were inserted into each of the prepared samples. The insertion depths below the soil surface at the tips of the SFDT and TEROS12 sensors were 75 mm and 55 mm, respectively. The output values were obtained four times for each sensor and sample. Two soil samples were taken near the sensor insertion points, and the θ of each sample was determined using the oven-drying method. The calibration equation for each sensor was obtained as a function of the relationship between the average of the obtained output values and θ.

### 2.3. Evaluation of Effects of EC and Air Gap Using Sand

The effect of EC on sensor outputs of the SFDT and TEROS12 sensors was evaluated using Toyoura sand. Sodium chloride (NaCl) solutions were prepared by mixing 2 g and 5 g of NaCl in 1 L of distilled water. The EC values of the solutions were 3.9 dS·m^−1^ and 9.2 dS·m^−1^, respectively. Sand samples were prepared by adjusting the water content in five steps using each solution. The samples were filled into the containers, and the output values were obtained by inserting the SFDT and TEROS12 sensors as described in Section 2.2.3.

The effect of air gap on sensor outputs was also evaluated. It is difficult to create air gaps of precise width between the soil and the probe. Therefore, a polyethylene tape with low dielectric constant was wrapped around the probes as a substitute for the air gaps. The dielectric constant of polyethylene at 25 °C is 2.37 [28]. The tape was uniformly wrapped around the entire probe of both of the sensors to a thickness of 0.42 mm. The tape-wrapped sensors were inserted into samples of Toyoura sand, and the output values were obtained; the water content was adjusted in two steps (θ = 0.086 m^3^ m⁻^3^ and 0.363 m^3^ m⁻^3^) using distilled water.

### 2.4. Theoretical Calibration Equation Model and Air Gap Model for SFDT Sensor

A theoretical model of the moisture calibration equation for the SFDT sensor was developed. The effect of the air gap was also modeled. We attempted to reproduce the experimental results using these models.

#### 2.4.1. Calibration Equation Model of Toyoura Sand

The theoretical calibration equation model of Toyoura sand with distilled water measured by the SFDT sensor was derived. Because the frequency range of the SFDT sensor was 1 to 6 GHz, it was necessary to consider the dielectric constant of free water in the microwave band. According to Debye’s relaxation model, the dielectric constant of free water ($\varepsilon_{fw}$) is

(6) $$\varepsilon_{fw}=\varepsilon_{\infty }+\frac{\varepsilon_{s}-\varepsilon_{\infty }}{1+j2\pi f\tau }+\frac{\sigma }{j2\pi f\varepsilon_{0}},$$

where $\varepsilon_{\infty }$ is the high-frequency dielectric constant, σ is the electrical conductivity, $\varepsilon_{0}$ is the permittivity of the free space, $\varepsilon_{s}$ is the static field dielectric constant, τ is the relaxation time, and f is frequency. $\varepsilon_{\infty }$ is 4.93, $\varepsilon_{s}$ is 78.36, and τ is 8.24 ps at about 25 °C [29]. The EC component in Equation (6) can be ignored because distilled water (σ=0) was used in the experiment. Because Toyoura sand does not include bond water ($\theta_{bw}=0$), the real part of the apparent dielectric constant ($\varepsilon^{\prime }$) is expressed as

(7) $$\varepsilon^{\prime }={\left[\left(1-\varphi \right)\varepsilon_{soil}^{\prime }{}^{\alpha }+\theta \varepsilon_{fw}^{\prime }{}^{\alpha }+\left(\varphi -\theta \right)\varepsilon_{air}{}^{\alpha }\right]}^{\frac{1}{\alpha }},$$

by applying to Equation (6) to Equation (4). Here, the imaginary part of the apparent dielectric constant ($\varepsilon^{\prime \prime }$) was obtained by Hilbert transform of $\varepsilon^{\prime }$ from Kramers–Kronig relations [30]. The apparent dielectric constant of the soil [31] $\varepsilon_{a}$ is

(8) $$\varepsilon_{a}=\frac{\varepsilon^{\prime }}{2}\left[1+\sqrt{1+{\left(\frac{\varepsilon^{\prime \prime }}{\varepsilon^{\prime }}\right)}^{2}}\right].$$

**Figure 4:**
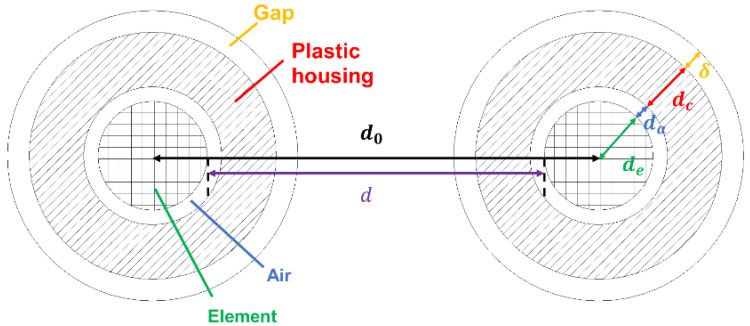
Cross-sectional view of the probe.

The actual prototype antennas were covered with a plastic housing as shown in Figure 4, for which there was a layer of the air between the antenna and the housing. $t_{pd}$ in Equation (2) is modified considering the structure of the probe as

(9) $$t_{pd}=\frac{1}{c}\left[\sqrt{\varepsilon_{a}}\left(d-2d_{a}-2d_{c}\right)+2d_{a}\sqrt{\varepsilon_{air}}+2d_{c}\sqrt{\varepsilon_{c}}\right],$$

where d is the distance between two antennas, $d_{a}$ is the thickness of the air clearance, $\varepsilon_{c}$ is the dielectric constant of the housing, and $d_{c}$ is the thickness of the housing. This is shown in Figure 4.

It was necessary to consider the radius of the element ($d_{e}$) because the propagation delay time ($t_{pd}$) did not include $t_{11}$ from Equation (1). Hence, d is $d_{0}-2d_{e}$, and the distance between the center of Tx and Rx antenna is $d_{0}$, as shown in Figure 4. The housing was made of polycarbonate, and $\varepsilon_{c}$ at 25 °C could be approximated as 2.78 [28]. The parameters of Toyoura sand in Equation (7) were taken as α=0.47, φ=0.42 [32], and $\varepsilon_{soil}^{\prime }=5$ [33].

#### 2.4.2. Air Gap Model for SFDT Sensor

Figure 4 also shows the model with a gap thickness (δ) on both antennas. The propagation delay time with the gap ($t_{w_{gap}}$) measured by the SFDT sensor is

(10) $$t_{w_{gap}}=\frac{1}{c}\left[\sqrt{\varepsilon_{a}}\left(d-2d_{a}-2d_{c}-2\delta \right)+2d_{a}\sqrt{\varepsilon_{air}}+2d_{c}\sqrt{\varepsilon_{c}}+2\delta \sqrt{\varepsilon_{gap}}\right],$$

where $\varepsilon_{gap}$ is the dielectric constant of the gap.

For comparison with the experimental values, the theoretical outputs ($t_{w_{gap}}$) were calculated by substituting the volumetric water contents (θ = 0.086 m^3^ m⁻^3^ and 0.363 m^3^ m⁻^3^) of the soil samples (see Section 2.3) into Equations (7) and (8) to obtain $\varepsilon_{a}$, before applying $\varepsilon_{a}$ to Equation (10). $\varepsilon_{gap}$ is the 2.37 value of polyethylene at 25 °C [28] to accommodate the experiment. The theoretical values of the air gap ($\varepsilon_{gap}$ = 1) were also calculated.

## 3. Results and Discussion

### 3.1. Water Content Calibration Using Sand

**Figure 5:**
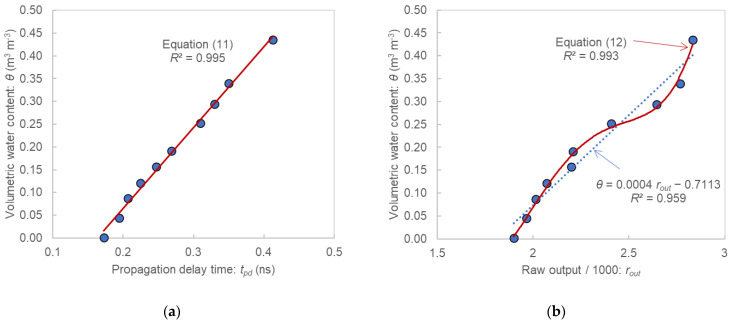
Relationships between the output and the volumetric water content of the samples: (**a**) for the SFDT sensor; (**b**) for the TEROS12 sensor.

The relationships between the output and θ of the samples are shown in Figure 5. The calibration equation of the SFDT sensor was

(11) $$\theta =1.772t_{pd}-0.2891.$$

The calibration equation of the TEROS12 sensor was

(12) $$\theta =2.1268r_{out}{}^{4}-19.025r_{out}{}^{3}+63.05r_{out}{}^{2}-91.31r_{out}+48.661.$$

The output of the SFDT sensor increased linearly with θ. When α was 0.5 in Equation (5), the plots exhibited linearity. According to the previous experiment, α of Toyoura sand was 0.47, which is close to 0.5 [32]. The coefficient of determination (*R*^2^) was greater than 0.99 even when a linear equation was used for calibration (Equation (11) in Figure 5a). 

Although the TEROS12 output also increased with increase in θ, linearity was lost in the high-water-content region, and *R*^2^ was 0.959 when the linear equation was used. When a quartic equation was used as the calibration equation, *R*^2^ improved to 0.993 (Equation (12) in Figure 5b). 

In general, a simpler calibration equation is more user-friendly. Therefore, the SFDT sensor is a user-friendly sensor because the relationship between the output and θ can be expressed by a linear equation with high accuracy. Incidentally, the output values of the SFDT sensor were extremely stable, with a maximum error of 1 ps ( ~0.002 m^3^ m−^3^) for each sample of the four measurements.

### 3.2. Evaluation of Effects of EC and Air Gap

**Figure 6:**
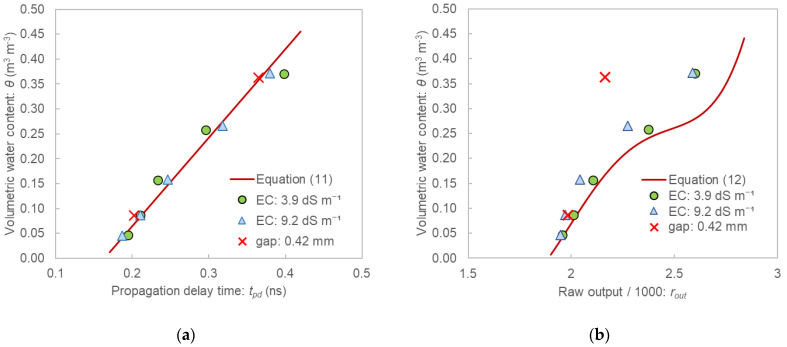
Relationships between the output and the volumetric water content when the saline samples were used (circle and triangle marks) and when the tape was wrapped around the sensors to simulate air gaps (cross marks): (**a**) for the SFDT sensor; (**b**) for TEROS12.

Figure 6 shows the relationships between the output and θ using saline samples, and polyethylene tape was wrapped around the probes to simulate air gaps. The calibration equations for the distilled water samples obtained in Section 3.1 (Equations (11) and (12)) are also shown in this figure. The plots of the saline samples of the SFDT sensor almost overlapped with the calibration equation even for the samples with pore water EC of 9.2 dS m^−1^. However, the TEROS12 sensor tended to underestimate θ in the saline samples. The degree of underestimation was particularly high in the high-water-content range. For example, substituting the output value obtained for the sample with EC of 9.2 dS m^−1^ and θ of 0.37 m^3^ m^−3^ into Equation (12) yielded θ of 0.28 m^3^ m^−3^, which means that the original θ was underestimated by 24 % (0.09 m^3^ m^−3^ in θ). The TEROS12 sensor was probably affected by EC owing to its use of low frequency (70 MHz). On the other hand, since the SFDT sensor used a higher frequency range (1 to 6 GHz) it was not affected by EC even at high salt concentrations.

The SFDT sensor only very slightly underestimated θ when tape was wrapped around the probe (Figure 6a). The reason the SFDT sensor was almost unaffected by the tape is because this sensor measures *ε,* which is the volume-weighted average of the dielectric constant of the soil and air gaps between the separated antennas; the tape width (0.42 mm) was sufficiently small compared to the distance between the antennas (23 mm). The TEROS12 sensor greatly underestimated θ in the high-water-content range (Figure 6b), which is probably because this sensor was affected by the low dielectric constant of the tape in the vicinity of the rods, as is the case with conventional TDR sensors.

### 3.3. Comparison of Theoretical Models and Experimental Results

**Figure 7:**
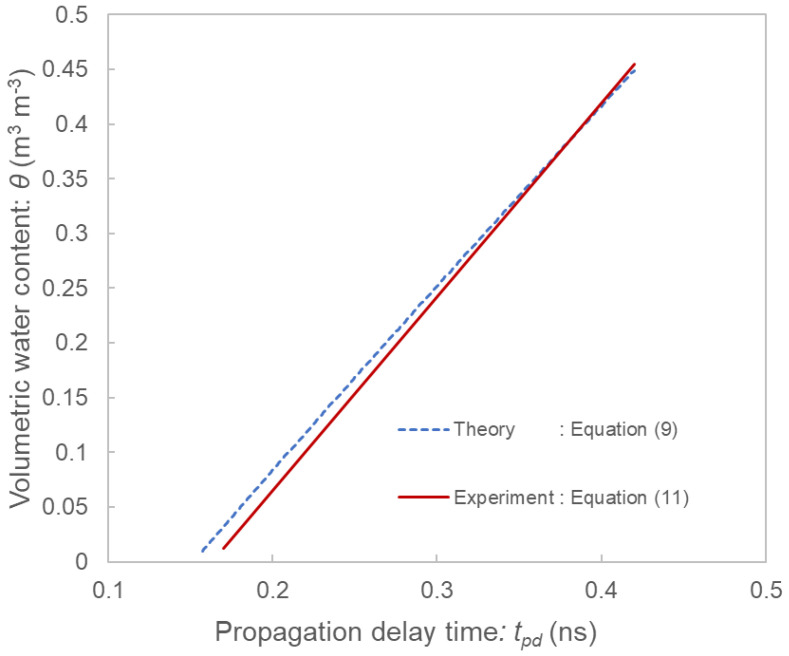
Comparison of the theoretical calibration equation from Equation (9) (blue line) and experimental calibration according to Equation (11) (red line) of the SFDT sensor using Toyoura sand.

The validity of the theoretical calibration equation model and the gap model were investigated by comparison with experimental results. Figure 7 shows the comparison of the theoretical calibration equation model from Equation (9) and the experimental calibration according to Equation (11) of Toyoura sand. The theoretical model agreed well with the experimental results.

**Figure 8:**
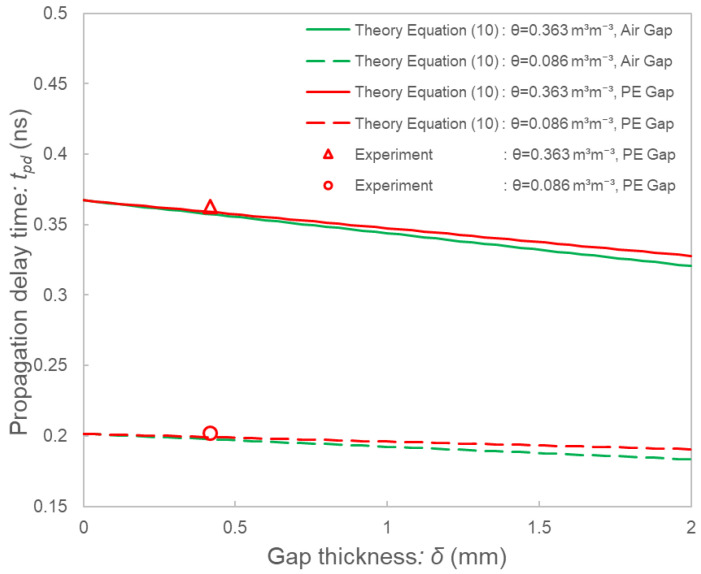
Comparison of the theoretical gaps models (lines) and experimental values (marks) of the SFDT sensor. The calculation values of the theoretical models of the polyethylene (PE) tapes are shown as red lines. The row data of $t_{w_{gap}}$ are plotted as experimental values (circle and triangle marks). The theoretical models of the air gap are also drawn for reference (green lines).

Figure 8 shows the comparison of the theoretical gap models and the experimental values of Toyoura sand. The theoretical models of the polyethylene (PE) gaps agreed with the experimental results, indicating that analytical approaches based on the theoretical models are possible for the SFDT sensor. According to the theoretical values, the output (propagation delay time) decreased with increasing gap thickness; however, the degree of decrease was slight. The theoretical values of the polyethylene gaps ($\varepsilon_{gap}$ = 2.37) and the air gaps ($\varepsilon_{gap}$ = 1) were close, indicating that the SFDT sensor is expected to be less affected by actual air gaps.

## 4. Conclusions

A new SFDT soil moisture sensor was developed, and the relationship between the output and θ, as well as the effects of air gaps and EC, were evaluated. Strong linearity was found in the relationship between the output and θ, and a first-order equation with high accuracy was employed as the calibration equation. Although the other commercially available sensors including TEROS12 were affected by salinity [8,11,12], the SFDT sensor was not affected by salinity even when the EC of the pore water was 9.2 dS m^−1^, which would represent a high salt concentration for irrigation water. In addition, the SFDT sensor was almost unaffected by air gaps, but the other sensors including TEROS12 significantly underestimated θ even for gaps below 0.5 mm [17]. The high linearity of the output of the SFDT sensor means that it is extremely accurate while being user-friendly; in addition, the small effect of EC and air gaps on the measured value indicates that it is highly suitable as a soil moisture sensor. Theoretical models of the SFDT sensor for the calibration equation and the air gaps were also developed. The models agreed well with the experimental results, indicating that analytical approaches are possible for the evaluation of the SFDT sensor.

The SFDT sensor, which is less affected by air gaps and EC, should be applied to soils where conventional sensors are difficult to apply, such as soils with poor rod adhesion, many macropores, or low density, as well as high-salt-concentration soils in arid regions. However, it may not be easy the sensor to insert into the soil because the probe volume is larger than that of other sensors. The developed SFDT sensor is a prototype, and further improvements will be necessary before it becomes commercially available, e.g., to reduce the volume, thereby improving insertion. Further evaluations using soils with different soil textures are also necessary, including theoretical approaches.

## Data Availability

Not applicable.
